# Mandibular Flexure and Its Significance: An In Vivo Cone Beam-Computed Tomography Proof-of-Concept Study

**DOI:** 10.3390/jcm12124149

**Published:** 2023-06-20

**Authors:** Jimmy Londono, Todd R. Schoenbaum, Alma Veronica Varilla Ortiz, Guillermo Franco-Romero, Vanessa Villalobos, Paolo Carosi, Eitan Mijiritsky, Alessandro Pozzi

**Affiliations:** 1 Ronald Goldstein Center for Esthetic and Implant Dentistry, Department of Restorative Sciences, Dental College of Georgia, Augusta, GA 30912, USA; jlondono@augusta.edu; 2 Department of Restorative Sciences, Dental College of Georgia, Augusta, GA 30912, USA; tschoenbaum@augusta.edu (T.R.S.); vvillalobos@augusta.edu (V.V.); 3 Independent Researcher, Puebla 72000, Mexico; alme_roni@hotmail.com; 4 Stomatology and Oral Rehabilitation Residency Program, Benemerita Universidad Autonoma de Puebla, Puebla 72000, Mexico; guillermo.franco@correo.buap.mx; 5 Department of Clinical Sciences and Translational Medicine, School of Dentistry, University of Tor Vergata, 00133 Rome, Italy; paolo.carosi@alumni.uniroma2.eu; 6 The Maurice and Gabriela Goldschleger School of Dental Medicine, Tel Aviv University, Tel Aviv 6997801, Israel; mijiritsky@bezeqint.net; 7 Head and Neck Maxillofacial Surgery, Tel Aviv Sourasky Medical Center, Sackler Faculty of Medicine, Tel Aviv University, Tel Aviv 6139001, Israel

**Keywords:** mandibular flexure, dental implants, CBCT, fixed dental prosthesis

## Abstract

The aim of this study was to assess intra-arch mandibular dimensional changes that may occur during mouth opening using cone beam-computed tomography (CBCT). Fifteen patients in need of any type of treatment whose execution considered a pre- and post-CBCT assessment consented and were enrolled. CBCTs were taken with the following settings: 90 kV, 8 mA, field of view (FOV) 140 by 100 mm (height and diameter), Voxel size 0.25 mm (high resolution). The pre-CBCT was executed in the maximum mandibular opening (MO), while the post-CBCT was in the maximum intercuspation (MI). A thermoplastic stent with radiopaque fiducial markers (steel ball bearings) was fabricated for each patient. Measurements were made using radiographic markers between contralateral canines and contralateral first molars and between ipsilateral canines and first molars on both sides. Paired *t*-tests were performed to evaluate the difference between open and closed positions on these four measurements. In the MO position were registered a significative tightening of the mandible at the canine (−0.49 mm, SD 0.54 mm; *p* < 0.001) and molar points (−0.81 mm, SD 0.63 mm; *p* < 0.001) and a significative shortening of the mandible on the right (−0.84 mm, SD 0.80 mm; *p* < 0.001) and left sides (−0.87 mm, SD 0.49 mm; *p* < 0.001). Within the study limitations, mandibular flexure determined a significant shortening and tightening between maximum intercuspation to maximum opening positions. Mandibular dimensional changes should be considered in light of other patient factors in the treatment planning of implant positioning and long-span complete arch implant-supported fixed prostheses in order to avoid technical complications.

## 1. Introduction

The flexure of the mandible (MF) is a multifactorial phenomenon that occurs simultaneously with mandibular movements and may determine changes in the shape of the mandible [1]. There are four patterns of jaw deformation in the human jaw: symphyseal bending, dorsoventral shear, body rotation, and anteroposterior cut [2]. According to Glanze et al., mandibular flexure is defined as “the change in jaw shape caused by the pterygoid muscles that contract during opening and protrusive movements” [3]. The strength exerted by these muscles plays a significant role in the flexure of the jaw, and the bending force is mainly exerted by the medial component of the obliquely located lateral pterygoid muscles [4]. The lower head of the lateral pterygoid muscle contributes to most of the mandibular deformation during opening [5]. Lateral pterygoid muscle contraction acts on the condyles that are pulled toward each other during the opening and protrusion movements [6,7]. Moreover, during opening, the lateral pterygoid muscles on both sides and the muscles of the floor of the mouth exert a force of contraction on the overall mandible [6]. The lateral pterygoid muscle was proven to contribute to the overall flexion of the mandible, especially in protrusion movements [8]. Due to differences in muscular strength for different facial patterns, brachyfacial subjects experienced greater deflection than dolichofacial subjects [9,10,11].

Previous studies reported increased biomechanical stress at the prosthetic and implant levels, poor passivity of fit, impression distortion, pain during function, de-cementation of the prosthesis, porcelain chipping, prosthetic screw loosening and fracture, bone resorption, and implant fracture [12,13,14,15,16,17,18,19,20]. Mandibular dimensional changes were investigated with transducers fixed on the tooth surface or implants, strain gauges, and intraoral optical surface scanning (IOS), as well as measuring plaster models and photographs at different stages of opening [21,22,23,24]. In vitro studies on stress distribution in the corpus of the mandible were performed using the photoelastic technique and finite element analysis (FEA) models [25]. Mandibular deformation was reported ranging from a few micrometers to around 1 mm [26,27]. Indeed, mandibular flexure may cause complications and failures in both conventional and implant-supported fixed dental prostheses (FDP), especially when a long-span prosthesis is planned to connect the anterior to the posterior region of the mandible [28]. Therefore, MF must be taken into account in the decision making related to the implant number, position, and prosthetic design to limit the potential surgical and prosthetic complications that may affect the surgical and prosthetic success of the implant treatment [17,29,30]. However, studies investigating MF were subject to many biases, mostly related to the difficulty of recording analogic or digital impressions in the MI position. The aim of this proof-of-concept study is to assess the dimensional changes determined by MF by means of cone beam-computed tomography (CBCT) taken at maximum intercuspation (MI) and maximum opening (MO) positions in order to better understand mandible 3D deformation due to masticatory muscle tension. The null hypothesis was that there is no significant difference in terms of length and width of the mandible between maximum intercuspation and maximum opening positions.

## 2. Materials and Methods

**Figure 1:**
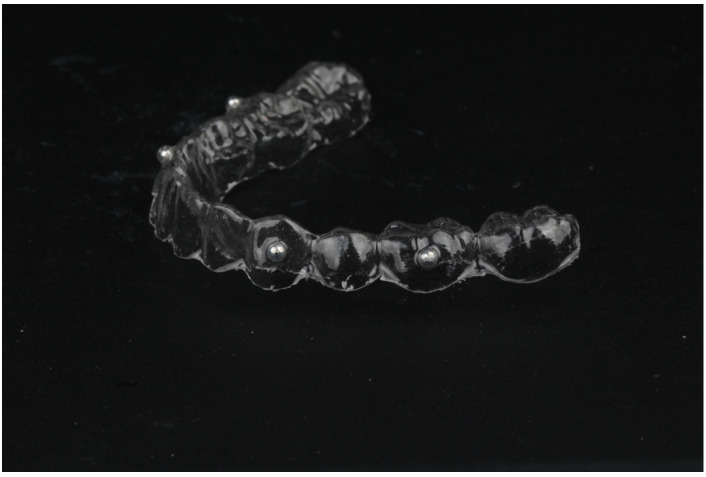
Thermoplastic stent with radiopaque fiducial markers (steel ball bearings) on the facial side.

**Figure 2:**
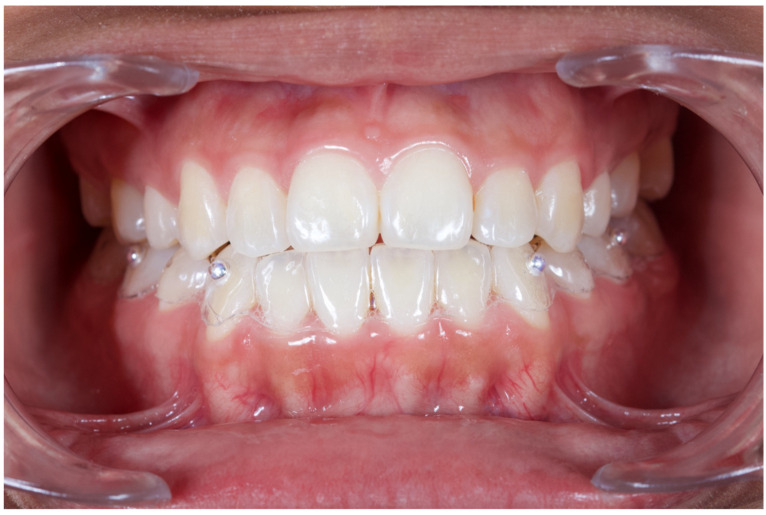
Standardized radiographic markers in position at canines and first molars prior to CBCT scan.

This proof-of-concept study fulfilled the Declaration of Helsinki as revised in 2013 and was reported in compliance with the STROBE guidelines [31]. The study protocol was revised and approved by the ethical committee of “Facultad de Estomatología—Benemérita Universidad Autónoma de Puebla” with identification code 2018065 and registered in ClinicalTrials.gov with the identifier NCT05718050. Fully dentate patients, 18 years of age or older, in good health (ASA I and II) were recruited among all the patients in need of being treated for any type of dental disease or treatments, such as bone regeneration procedures, that consider a pre-CBCT examination and a post-operative follow-up with CBCT. Participants were recruited and signed the informed consent after being properly educated about the nature and the investigating procedures of the study [32]. Exclusion criteria included: active periodontitis with tooth mobility, absence of lower canines and or first molars, metal-based FDP in the canine or molar areas, neuromuscular disorders, temporomandibular joint disorders, parafunctional habits, any systemic condition preventing surgery, history of oro-maxillofacial radiation therapy, history of drug or alcohol abuse, heavy smoking, uncontrolled diabetes, pregnancy, or lactation. All the patients were screened and prepared according to a strict study protocol. A mandibular silicon impression was taken, and a vacuum-molded thermoplastic stent with radiopaque fiducial markers (steel ball bearings 4 mm Ø) on the facial side of the canines and molars was fabricated for each patient (Figure 1 and Figure 2).

**Figure 3:**
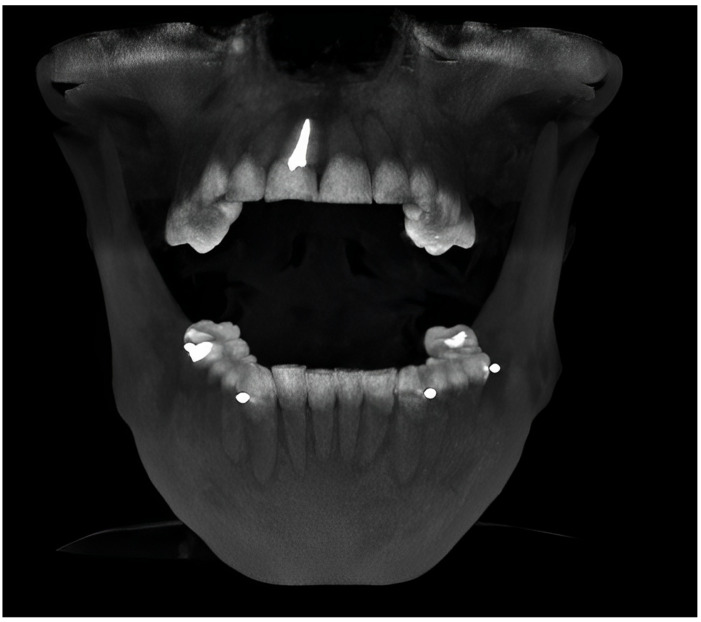
The pre-CBCT was executed in maximum opening position.

**Figure 4:**
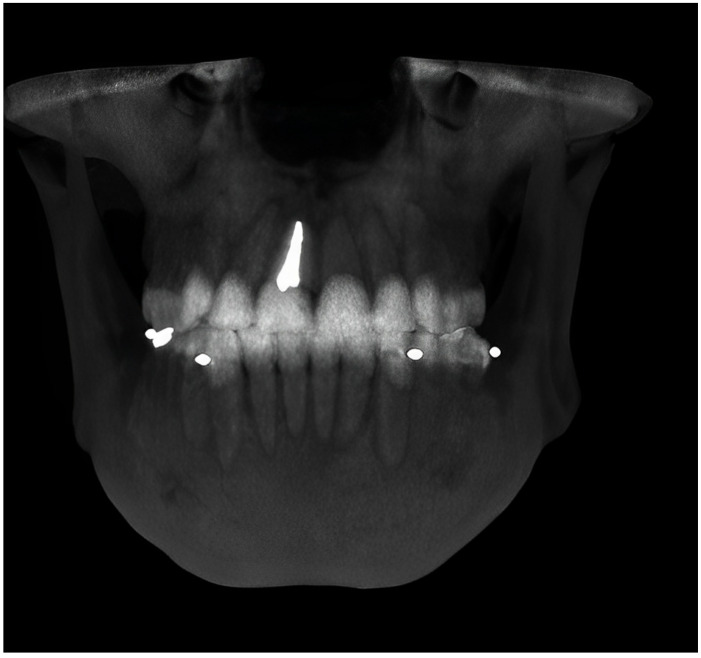
The post-CBCT was executed in maximum intercuspation (MI).

Two cone beam-computed tomographs (CBCTs) were made per patient with their radiographic stent properly fitted and secured on the remaining dentition at the MI and MO positions at the University Advanced Digital Diagnostic Center (Figure 3 and Figure 4).

The CBCT examination was performed with a large field of view (FOV) detector computed tomography (SCANORA 3DX; Dexis LLC, Pennsylvania Avenue NW, Suite 300, Washington, DC, USA) to avoid any artifacts related to any software algorithm stitching procedure and using the following setting: 90 kV, 4 mA, and 140 by 100 mm (height and width) FOV to decrease the radiation dose without affecting the quality of the examination [33]. A blind assessor made 8 measurements per patient on a 3D planning software (DTX Studio Implant software, Dexis LLC, Washington, DC, USA). To test the assessor measurements agreement and consistency, four random CBCTs of included patients were measured again after two weeks. The intraclass correlation coefficients (ICCs) were 0.97 (95% CI: 0.95 to 0.99; *p* < 0.001) and 0.98 (95% CI: 0.95 to 0.99; *p* < 0.001), reporting acceptable consistency and reliability. The measurements were executed on the cross-section radiographic reconstructions of the patient’s anatomy in MO and MI, measuring the distance between the radiopaque fiducial landmarks at the contralateral canines and contralateral molars and between ipsilateral canines and molars on both sides. Each distance between the fiducial landmarks was recorded in mm, and the differences between the same distances in maximum opening and maximum intercuspation were analyzed for each patient in order to obtain linear mean changes (mean + standard deviation) in width from right molar to left molar from right canine to left canine (molar to molar, canine to canine) and in length from right canine to right molar and from left canine to left molar (canine to molar right and left). Moreover, paired *t*-tests were performed in JMP Pro 15 (SAS institute) to test for statistically significant differences between open and closed jaw positions.

## 3. Results

Fifteen patients (mean age 49.7 SD 11.3 years, range 27 to 76) were eligible and enrolled in the study. Each patient underwent 2 CBCT examinations (in MI and MO positions), and in total, 30 Digital Imaging and Communications in Medicine (DICOM) sets of files were analyzed. A total of 120 measurements were executed and analyzed (Table 1).

In terms of width, the mean difference from molar to molar was −0.81 mm (SD 0.63 mm), and from canine to canine was −0.49 mm (SD 0.54 mm). In terms of length, the mean difference from canine to molar on the right side was −0.84 mm (SD 0.80 mm), while on the left side was −0.87 mm (SD 0.49 mm). The paired *t*-test revealed a statistically significant tightening at the molar point (*p* = 0.00009) and at canine points (*p* = 0.00178). The shortening was significant both on the right side (*p* = 0.00062) and on the left side (*p* = 0.000004). The results are summarized in Table 2.

## 4. Discussion

Within the limitations of the present study, the mandible experienced a statistically and clinically significant shortening and tightening from the maximum intercuspation to maximum opening positions. Therefore, the null hypothesis that there is no significant difference in the extent of mandibular dimension in length or width between closed and open jaw positions was rejected. The main limitation of the present study is the lack of a priori sample size calculation. However, to the best of the authors’ knowledge, this is the first study investigating the intra-arch dimensional changes of the mandible that occur during mouth opening in dentate patients using a CBCT, bypassing most of the bias related to previously published methods of assessment. Thus, this proof-of-concept study design can be considered a pilot study for future observational studies with a larger sample size and comparison between different facial, skeletal, and muscular patterns [23]. Nevertheless, 15 patients were investigated with a CBCT examination, and a total of 120 measurements were executed and analyzed following a strict protocol without deviations. Consequently, preliminary and generalizable conclusions could be drawn.

The stiffness of the mandible is likely influenced by the cross-sectional area, the cortical thickness, the characteristics of the bone, and, in particular, the amount of cancellous bone and the shape of the mandible [1]. Age-related bone loss is associated with reduced density and mineral content of cortical and cancellous bone. With increasing age, the mandible becomes more porous and more prone to flexure [17,22]. On the contrary, the flexibility of bone tissue decreases with age due to the decrease in collagen-related bone elasticity. Mandibular flexure was not related to sex [34]. A recently published systematic review by Mijiritsky et al. [35] reported that MF was mainly experienced during protrusion and maximum opening movements and must affect the clinical decision making related to the whole prosthodontic treatment, starting from the impression technique to the choice of different types of restorations and materials.

A recently published study investigating the mandibular flexure phenomenon on width using an intraoral optical surface scanner reported a linear increase in the mean dimensional changes from the anterior to the posterior region ranging from 0.048 to 0.630 mm and from 0.089 to 0.710 mm in dentate and edentulous patients, respectively [23]. Such a difference was not statistically meaningful between the two groups. It was reported that there was no change in mandible width up to 28% of the maximum opening, and the change in width is directly related to the size of the mouth opening [7]. However, all the studies that investigated the mandibular changes, taking a conventional or an optical surface scanning digital impression, were biased by the position of the mandible, which could not be the maximum intercuspation because of the need to insert into the mouth the impression trays or the scanner tip (about 20 mm), and this needs an opening bigger than the aforementioned 28% [23,24]. Conventional impressions required several movements of the mandible to insert and remove the impression trays. Moreover, conventional impression materials were subject to elastic deformations when they were removed from the patient’s mouth. Digital impressions overcome this issue by bypassing the impression materials. Even if there is no elastic deformation, the images recorded by the scanner are subject to stitching phenomenon that may result in a non-accurate or deformed 3D impression caused by the superimposition of several images captured by the scanner.

This may explain the experienced differences in the mandibular dimensional changes with the data reported in the present study. The authors reported a significative tightening of the mandible at the canine (−0.49 mm; *p* < 0.001) and molar points (−0.81 mm; *p* < 0.001) and a significative shortening of the mandible on the right (−0.84 mm; *p* < 0.001) and left sides (−0.87 mm; *p* < 0.001).

In light of such potentially clinically relevant tightening and shortening, the mandibular flexure phenomenon should be deeply considered when a conventional or implant-supported long-span mono-lateral or bilateral FDP is planned, particularly when it is extended to the posterior quadrants (first and even more second molar). Patient-related factors such as the anatomic characteristics of the bone (cortical–spongious ratio), mandible size, muscle strength, and maximum opening should be evaluated because such factors might affect the amount of flexure and, thereafter, the success and prognosis of the dental prosthesis [1,21,35]. Moreover, in the case of conventional FDP, the stress created by flexure of the mandible is totally absorbed by the periodontal ligament and may cause periodontal enlargement and teeth mobility.

The amount of tightening and shortening experienced in the study and caused by the MF phenomenon may be of paramount importance in implant treatment, particularly in the case of a rigid long-span screw-retained prosthetic superstructure and may concentrate at the bone and prosthetic levels high-stress gradients due to jaw deformation [1]. Such increased strains and stress at the implant prosthetic complex may increase the biological and biomechanics complications affecting the overall implant and prosthetic success. Medium mandibular deflection poses challenging problems for both conventional prostheses and implants. Biological and prosthetic complications related to high stress at the bone and prosthetic interface may cause loosening and fracture of the implant or prosthetic screws, chipping of the veneering material, and bone resorption around the implant [9,10,11,12,13,14,15,16,17]. Because of this situation, it is vitally important to contemplate it, try not to decrease its gap amplitude, and thus achieve successful, predictable treatments with suitable longevity. Therefore, excessive build-up of tension at the implant and prosthetic interfaces could affect the outcome of implant treatment and rehabilitation [19].

Moreover, extreme movements of the mandible in protrusion and maximum opening should be avoided by the patient rehabilitated with long-span FDP because the maximum medial deflection has been determined at the maximum opening of the mouth [20].

Patients with osteoporosis, bone deformities, or poor bone density tend to have increased mandibular deflection, and aged edentulous patients with smaller symphyseal areas are more prone to jaw distortion during the movements; thus, the mandibular deflection may affect the success of implant treatment since the osseointegration time and lead to bone loss around the implants and implant failure [17]. Hobkirk and Schwab measured force transmission between osseointegrated (OI) implants in the premolar regions of the edentulous mandible using intraoral transducers linked to OI implants, experiencing deformations of up to 420 microns and force transmission of up to 16 N as a result of jaw movement from the rest position. Greater displacements and forces were observed in active opening and protrusion than in lateral excursions. There were wide variations from subject to subject, and while the effects of these phenomena are not known, they may be potentially harmful to the interfaces between the implants and bone and the various components of the implant superstructure [36,37,38].

Lindquist et al. measured bone loss associated with osseointegrated implants that were placed between the mental foramen and restored with a complete arch FDP with posterior cantilevers. The results showed that a greater crestal bone loss around the posterior implants than the anterior ones placed in the symphysis region may be potentially caused by mandibular deflection [18].

Therefore, it may be advisable to reduce the cantilever length or sectioning of the hybrid prosthesis in the middle line to relieve stress and improve the longevity of the prosthesis. It may also be that splinting implants with a sufficiently robust fixed FDP reduce the flexure of the mandible, limiting the potential drawbacks. Pozzi et al. [30] reported high prosthetic survival, stable bone levels, and low peri-implantitis rates in long-span, screw-retained, fixed, zirconia FDPs with a follow-up to 12 years using large connectors (cross-sectional area mean, SD 17 ± 1.11 mm^2^). On the contrary, if the prosthetic framework is insufficiently rigid or robust, it may be at a much higher risk of fracture or delamination of any overlying porcelain materials or any other prosthetic complications. The position of the implants in the mandible should be carefully planned. The present study reported a mean tightening of the mandible between the canines of 0.49 mm and 0.89 mm between the molars. In order to counteract the significative tightening of the mandible, Mijiritsky et al. [35] suggested splitting the complete arch implant-supported prosthesis into two or three segments. As an alternative, placing the implants mesial to the mental foramen could be a valid option to rehabilitate complete arches preventing biological and prosthetic complications over the years. A recent study by Agliardi et al. [39] reported a high prosthetic survival rate (98.01%) in the mandible after 16 years of function. The investigated prostheses were supported by four implants, two straight and two tilted mesially to the mental foramen, avoiding placing implants in the molar area. With this implant distribution, flexure of the mandible may not influence the overall surgical and prosthetic survival of the implant treatment, even if a one-piece prosthesis was realized.

As a matter of fact, comprehensive digital planning of the implant positioning and design of definitive FDP according to all the patient-related factors that may enhance MF, such as the vertical face aspect, the structure of the mandible symphysis, and bone density and thickness, could significantly decrease the possibility of complications over the years [30,31,32,33,34,35,36,37,38,39,40,41].

In individual patients undergoing full arch mandibular treatment, the clinician should consider and weigh the patient-specific factors that might contribute to increased jaw deformation and its propensity to cause complications or failures. If a patient has a brachycephalic face pattern, strong musculature, reduced vertical mandibular bone heights, and decreased bone density, the forces deforming the jaw may overcome the stabilizing effect of a framework or the preload of the prosthetic implant screws. In this patient scenario, the clinician should consider placing all the implants in a more anterior position between the two mental foramina, where the mandibular flexure phenomenon is less pronounced.

## 5. Conclusions

Within this proof-of-concept study limitations, mandibular flexure determined a significant shortening and tightening from maximum intercuspation to maximum opening position in dentate patients. Such dimensional changes should be considered in light of other patient factors to execute more comprehensive digital planning since implant positioning to definitive FDP design. Limiting long-span complete arch-fixed prostheses supported by distal implants in molar positions may decrease the occurrence of complications over the years.

## Figures and Tables

**Table 1:** Results for each patient after the analysis in millimeters (mm). (M-M molar to molar (width)); C-C (canine to canine (width)); C-M RIGHT (canine to molar on the right side (length)); C-M LEFT (canine to molar on the left side (length)); maximum opening (MO); maximum intercuspation (MI).

Width Variations					Length Variations			
#	M-M MO	M-M MI	C-C MO	C-C MI	C-M RIGHT MO	C-M RIGHT MI	C-M LEFT MO	C-M LEFT MI
1	47.52	48.67	19.95	20.5	23.88	24.67	24.27	24.77
2	48.74	48.94	23.99	24.3	28.98	28.18	22.55	22.77
3	45.85	47.93	29.21	28.87	23.33	23.68	25.22	26.47
4	44.37	44.69	24.21	24.82	23.58	23.6	22.45	23.26
5	43.77	44.77	23.53	24.92	23.77	24.52	24.14	25.62
6	54.78	54.91	25.83	27.18	23.04	24.09	24.59	25.53
7	45.32	47.43	26.41	27.19	26.51	26.37	24.71	25.49
8	45.32	45.84	24.5	25.23	23.24	25	23.94	24.92
9	56	56.49	36.43	36.8	22.28	22.67	9.81	10.61
10	55.4	56.06	26.72	27.03	9.45	11.23	24.1	24.31
11	55.41	55.81	32.01	31.23	15.09	16.94	12.99	13.08
12	55.09	55.61	31.68	32.11	12.61	14.56	11.86	13.15
13	53.2	54.41	30.61	31.26	14.8	15.45	14	15.66
14	52.8	53.4	30.71	31.31	15.53	16.93	13.09	13.7
15	52.41	53.4	32.2	32.6	24.98	25.79	9.51	11.06

**Table 2:** Dimensional change in the mandible from maximum opening (MO) to maximum intercuspation (MI).

Mandibular Dimensional Change (*n* = 20)	Molar–Molar Width	Canine–Canine Width	Canine–Molar Length (Right Side)	Canine–Molar Length (Left Side)
Mean difference (MO—MI; mm)	−0.81	−0.49	−0.84	−0.87
Std Dev	0.62	0.54	0.80	0.49
95% CI	(−1.16, −0.46)	(−0.79, −0.18)	(−1.28, −0.39)	(−1.15, −0.60)
*p*-Value	0.00009	0.00178	0.00062	0.000004

## Data Availability

Data of the present study can be available upon reasonable request from the corresponding author.
